# Annotation of rule-based models with formal semantics to enable creation, analysis, reuse and visualization

**DOI:** 10.1093/bioinformatics/btv660

**Published:** 2015-11-11

**Authors:** Goksel Misirli, Matteo Cavaliere, William Waites, Matthew Pocock, Curtis Madsen, Owen Gilfellon, Ricardo Honorato-Zimmer, Paolo Zuliani, Vincent Danos, Anil Wipat

**Affiliations:** ^1^Interdisciplinary Computing and Complex BioSystems Research Group, School of Computing Science and Centre for Synthetic Biology and the Bioeconomy, Newcastle University, Newcastle upon Tyne, UK,; ^2^School of Informatics, University of Edinburgh, Edinburgh, UK and; ^3^Turing Ate My Hamster Ltd., Newcastle upon Tyne, UK

## Abstract

**Motivation:** Biological systems are complex and challenging to model and therefore model reuse is highly desirable. To promote model reuse, models should include both information about the specifics of simulations and the underlying biology in the form of metadata. The availability of computationally tractable metadata is especially important for the effective automated interpretation and processing of models. Metadata are typically represented as machine-readable annotations which enhance programmatic access to information about models. Rule-based languages have emerged as a modelling framework to represent the complexity of biological systems. Annotation approaches have been widely used for reaction-based formalisms such as SBML. However, rule-based languages still lack a rich annotation framework to add semantic information, such as machine-readable descriptions, to the components of a model.

**Results:** We present an annotation framework and guidelines for annotating rule-based models, encoded in the commonly used Kappa and BioNetGen languages. We adapt widely adopted annotation approaches to rule-based models. We initially propose a syntax to store machine-readable annotations and describe a mapping between rule-based modelling entities, such as agents and rules, and their annotations. We then describe an ontology to both annotate these models and capture the information contained therein, and demonstrate annotating these models using examples. Finally, we present a proof of concept tool for extracting annotations from a model that can be queried and analyzed in a uniform way. The uniform representation of the annotations can be used to facilitate the creation, analysis, reuse and visualization of rule-based models. Although examples are given, using specific implementations the proposed techniques can be applied to rule-based models in general.

**Availability and implementation:** The annotation ontology for rule-based models can be found at http://purl.org/rbm/rbmo. The krdf tool and associated executable examples are available at http://purl.org/rbm/rbmo/krdf.

**Contact:** anil.wipat@newcastle.ac.uk** or **vdanos@inf.ed.ac.uk

## 1 Introduction

The last decade has seen a rapid growth in the number of model repositories (Li *et* *al.*, 2010; Misirli *et* *al.*, 2014; Moraru *et al.*, 2008; Snoep and Olivier, 2003; Yu *et* *al.*, 2011). Creating models and populating these repositories is not a trivial task as it requires expert knowledge and integration of different types of biological data from multiple sources (Endler *et* *al.*, 2009). Classically, these data are used to derive the structure of, and parameters for, models. However, biological data can also be used to annotate models and their components. These annotations act as metadata to decorate a model with links to biologically relevant information (Blinov *et* *al.*, 2010). Machine-readable annotations are also important to facilitate the automated exchange, reuse and composition of complex models from simpler ones. As the number and size of models increase, the availability of informative annotations becomes more important. Annotation techniques can then be applied to rule-based models that can represent in a compact way the complexity inherent in biological systems (Blinov *et al.*, 2008; Danos and Laneve, 2004).

Rule-based languages, such as Kappa (Danos and Laneve, 2004; Danos *et* *al.*, 2007) and BioNetGen (Faeder *et* *al.*, 2009), have emerged as helpful tools for modelling biological systems (Köhler *et* *al.*, 2014). Rule-based modelling is widely used to concisely represent the combinatorial explosion of the state space inherent in modelling biological systems. Rule-based models comprise agents representing biological molecules and rules representing biological interactions between agents. These rules are sufficient to allow models to be simulated, but the biological meanings of the model entities are not directly accessible. These languages do have facilities for comments that are intended for unstructured documentation directed at the modeller or programmer. However, these comments are not computationally accessible. Currently, there is no standardized syntax to store annotations within models written in rule-based languages.

Model annotation has already been widely applied in reaction-based models. For example, Saint has been developed to enrich models by identifying and integrating biological information (Lister *et* *al.*, 2009) in some cases fruitfully leading to new discoveries (Lister *et* *al.*, 2010). Based on existing model annotations, this tool can suggest the addition of new entities to extend models. Annotations can also be used to verify and merge models, and to check for inconsistencies (Krause *et* *al.*, 2010). Moreover, model repositories can be searched using commonly used annotation terms. BioModels (Li *et* *al.*, 2009, 2010) is a repository of models and, at the time of writing, includes 1379 models, 583 of which are manually annotated (http://www.ebi.ac.uk/biomodels-main/). These annotations can be used by tools such as ReactionFinder (Neal *et* *al.*, 2014) to search for reactions that can be reused as modular components of larger models.

Model annotation is an ongoing research topic in synthetic biology. The Virtual Parts Repository (Misirli *et* *al.*, 2014) is a repository of modular models of biological parts. Models in this repository are defined with inputs and outputs, which are annotated semantically. These annotations make the models computationally composable and facilitate the model-driven design of biological systems. When these models are annotated with additional information such as nucleotide sequences and types of biological parts, the resulting composed models can act as blueprints to derive synthetic biological systems (Misirli *et* *al.*, 2011; Roehner and Myers, 2013).

Annotations can also be used to aid in the computational conversion of models into a variety of other data formats. For example, PDF documents (Li *et* *al.*, 2010) or visual graphs (Funahashi *et* *al.*, 2007) can be automatically generated from annotated models in order to aid human understanding. Annotations can also help in the provision of the extra information necessary to convert between modelling formalisms (Blinov *et* *al.*, 2008).

### 1.1 Rule-based models

**Fig. 1.**
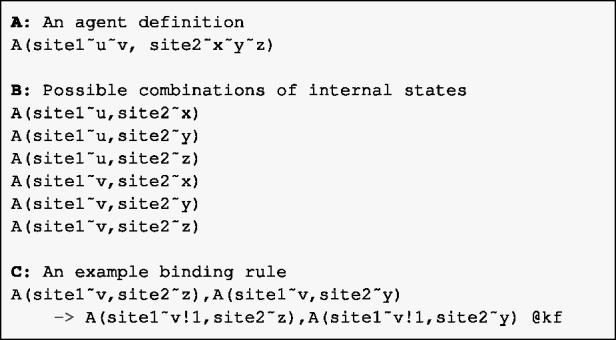
(**A**) An agent with two sites. site1 has two possible internal states while site2 has three. (**B**) This agent can be used in six different ways depending on the internal states of its sites. (**C**) A rule that specifies how agent A forms a dimer when the state of site1 is v and the states of site2 are z and y, respectively. The notation !n means that the sites where it appears are bound together. The constant kf denotes the kinetic rate associated with the rule

Biological entities are represented by agents in Kappa and molecule types in BioNetGen (we shall use ‘agent’ to generically refer to both agents and molecule types in this paper). In general, agents may include any number of sites that represent the points of interactions between agents. For example, the binding domain site of a transcription factor (TF) agent can be connected to a TF binding site of a DNA agent. Moreover, sites can have states. For instance, a TF could also have a site for phosphorylation and the DNA binding can be constrained to occur only when the state of this site is phosphorylated. For an agent with two sites, of which one with two internal states and the other with three, the number of possible combinations is six (Fig. 1A, B). A pattern is an (possibly incomplete) expression of an agent in terms of its internal and binding states. Rules, that specify biological interactions, consist of patterns on the left hand side which, when match, produce the result on the right hand side (Fig. 1C). Specific patterns of interest can be declared as an observable of the model.

The need for annotations in rule-based languages has already been acknowledged. Chylek and co-workers proposed guidelines for visualizing and annotating models (Chylek *et* *al.*, 2011). Although the authors suggest extending rule-based models to include metadata, their study focuses upon documenting models with biological information using comments to aid the understanding of models for humans. Additionally, PDF documents, called model guides, are made available. Using a similar approach, a model guide for a large rule-based model has also been demonstrated in the form of a wiki (Creamer *et* *al.*, 2012). These guides include graphs, depicting interactions of agents through rules, which are enriched with further biological information. Creating a model guide is a manual process and may not be time-efficient for large models. Recently, Klement and co-workers demonstrated embedding more structured comments into rule-based models (Klement *et* *al.*, 2014). Data are added in the form of property/value pairs using a specific syntax; however, this study also focuses on presenting data for humans.

Machine readable annotations have been applied to rule-based models using PySB, a programming framework for writing rules using Python (Lopez *et* *al.*, 2013). A model object in PySB includes lists of agents and rules and also a list for machine-readable annotations. However, this approach is insufficiently general. Annotations cannot be applied to sites, states or subrules. PySB is a framework written in the Python programming language and requires running a program to generate rulesets for the simulators. This means that any processing of the annotations must also be written in or have facilities for interpreting Python, and furthermore that users must program their models in Python which is not always desirable (Chylek *et* *al.*, 2014).

### 1.2 Annotating rule-based models

Model annotation has already been widely applied in different modelling formalisms. Existing annotation standards and approaches can also be used in rule-based models by taking care of their specific needs. Rule-based models are usually written in textual formats, in which agents and rules are defined in single lines (Danos *et* *al.*, 2007). In general, the syntactic definition of an agent identifies sites and states in rule-based models but the semantics of sites and states is usually clear only to the modeller. For machine access, this information must be exposed in a structured way. Moreover, it is not straightforward to map sites and states to annotations directly, since these entities are part of agents and not top level modelling entities. Additionally, it is often desirable to annotate a specific pattern with a particular subset of sites and states. Therefore, patterns should also be annotatable. The issue of mapping annotations may also occur for rules that contain subrules (for example, as part of Kappa hybrid rules, additional rules can be defined). A subrule does not correspond to a single entity so it is difficult to unambiguously refer to in order to link biological information. Therefore, we extend the syntax of rule-based models to incorporate annotations.

Existing metadata resources include machine readable controlled vocabularies and ontologies, Web services providing standard access to external identifiers and guidelines for the use of these resources. For example, the Minimum Information Requested in the Annotation of Models (MIRIAM) standard (Le Novere *et al.*, 2005) was proposed in order to standardize the minimal information required for the annotation of models. In this proposal entities in mathematical models are linked to external information through the use of unique Uniform Resource Identifiers (URIs), which are embedded within models and can be used to retrieve such information. The uniqueness and global scope of these URIs are important for *disambiguation* of model agents, variables and rules.

Annotations are composed of statements. A statement can link a modelling entity to a value using a standard qualifier term, which represents the relationship between the entity and the value. These qualifiers often come from controlled vocabularies or ontologies in order to unambiguously identify the meaning of modelling entities. URIs are used as values to link these entities to external resources, and hence to a wealth of biological information by keeping the amount of annotations minimal. The links themselves are typed, again with URIs. The qualifiers and resources that they refer to are typically drawn from ontologies that encode a Description Logic (http://www.w3.org/TR/owl-features) for a particular domain.

#### 1.2.1 Unifying semantics

There are several metadata standard initiatives that provide controlled vocabularies from which standard terms may be drawn. For example, metadata terms provided by the Dublin Core Metadata Initiative (DCMI) (http://www.dublincore.org/documents/dcmi-terms) or BioModels qualifiers can be used to describe modelling and biological concepts (Le Novère and Finney, 2005; Li *et* *al.*, 2010). Ontologies such as the Relation Ontology provide formal definitions of relationships that can be used to describe modelling entities (Smith *et* *al.*, 2005). There are also several other ontologies and resources that are widely used to classify biological entities represented in models with standard values (Swainston and Mendes, 2009): the Systems Biology Ontology (SBO) (Courtot *et* *al.*, 2011) to describe types of rate parameters; the Gene Ontology (GO) (The Gene Ontology Consortium, 2001) and the Enzyme Commission numbers (Bairoch, 2000) to describe biochemical reactions; the Sequence Ontology (SO) (Eilbeck *et* *al.*, 2005) to annotate genomic features and unify the semantics of sequence annotation; the BioPAX ontology (Demir *et* *al.*, 2010) to specify types of biological molecules and the Chemical Entities of Biological Interest (ChEBI) (Degtyarenko *et* *al.*, 2008) terms to classify chemicals. URIs of entries from biological databases, such as UniProt (Magrane and UniProt Consortium, 2011) for proteins and KEGG (Kanehisa *et* *al.*, 2008) for reactions, can also be used to uniquely identify modelling entities.

#### 1.2.2 Unifying data access

Accessing external resources through URIs can also be standardized using MIRIAM or Identifiers.org URIs (Juty *et* *al.*, 2012), although the former is not directly resolvable and requires out of band knowledge to retrieve information. These URIs consist of collections and their terms, which may represent external resources and their entries respectively. For example, the MIRIAM URI urn:miriam:uniprot:P69905 (a dereferencable URI using the MIRIAM Web services is http://www.ebi.ac.uk/miriamws/main/rest/resolve/urn:miriam:uniprot:P69905) and the Identifiers.org URI http://identifiers.org/uniprot/P69905 can be used to link entities to the P69905 entry from UniProt. The relationships between modelling entities, annotation qualifiers and values can be represented using the Resource Description Framework (RDF) (http://www.w3.org/TR/rdf-syntax-grammar) graphs.

#### 1.2.3 Unifying syntax

RDF represents knowledge in the form of (subject, predicate, value) triples, in which the subject can be an anonymous reference or a URI, the predicate is a URI and the object can be a literal value, an anonymous reference or a URI. Subjects and objects may refer to an ontology term, an external resource or an entity within the model. RDF graphs can be serialized in different formats such as XML or the more human readable Turtle format (http://www.w3.org/TR/turtle). Modelling languages such as the Systems Biology Markup Language (SBML) (Hucka *et* *al.*, 2003), CellML (Cuellar *et* *al.*, 2003; Hedley *et* *al.*, 2001) and Virtual Cell Markup Language (Moraru *et* *al.*, 2008) are all XML-based and provide facilities to embed RDF/XML annotations (Endler *et* *al.*, 2009). There are also other exchange languages, such as BioPAX and the Synthetic Biology Open Language (SBOL) (Galdzicki *et* *al.*, 2012, 2014), that can be serialized in RDF/XML allowing custom annotations to be embedded.

In this paper, we extend the use of RDF and MIRIAM annotations for rule-based models. We describe a syntax to store machine-readable annotations and an ontology to facilitate the mapping between rule-based model entities and their annotations. Annotations are then illustrated using terms from this ontology and some examples of their use provided.

## 2 Annotation approach for rule-based models

### 2.1 Syntax for storing annotations

A common approach, when trying to add additional structured information to a language where it is either undesirable or infeasible to change the language itself, is to define a special way of using comments. This practice is long established for structured documentation or ‘docstrings’ in programming languages (Acuff, 1988) (https://www.gnu.org/prep/standards) and has been used for extending otherwise fixed data formats since punch cards were current technology (Buneman, 2015). We adopt this approach so that models written using the conventions that we describe here do not require modification of the modelling software, KaSim (https://github.com/Kappa-Dev/KaSim) and RuleBender (Xu *et* *al.*, 2011), that is their primary target.

We use the language’s comment delimiter followed by the ‘^’ character to denote annotations in the textual representation of rule-based languages. Kappa and BioNetGen use the ‘#’ symbol to identify comment lines, so in the case of these languages, comments containing annotations are signalled by a line beginning with ‘#^’. This distinguishes between comments containing annotations and comments intended for human consumption. Annotation data for a single modelling entity or a model itself can be declared over several lines and each line is prefixed with the ‘#^’ symbol.

### 2.2 Annotation format

Annotations are serialized in the RDF/Turtle format. This representation balances the need for a machine-readable syntax and a human readable textual representation. As the rule-based modelling languages that we are annotating are themselves structured text formats, RDF/Turtle is more suitable than the XML-based representations of RDF.

Annotations for a single rule-based model entity are simply a list of statements. Annotations may refer to other annotations within the same model. When all the lines corresponding to a rule-based model and the annotation delimiter symbols are removed, the remaining RDF lines represent a single RDF document. This enables annotations to be quickly and easily extracted without special tools (for example, on a UNIX system, the following pipeline can be used: grep fl^#\^fl— sed fls/^#\^//fl).

### 2.3 Mapping between entities and annotations

XML-based modelling languages such as SBML and CellML already provide opening and closing tags, and annotations are encapsulated within the definition of a modelling entity. In textual rule-based models, it is difficult to store annotations within a modelling entity since Kappa and BioNetGen represent modelling entities such as agents and rules as single lines of text. As a result, there is no natural location to attach annotations to an entity. Here, we propose to achieve the mapping between a modelling entity and its annotation by defining an algorithm to construct a URI from the symbol used in the modelling language. The algorithm used in this paper generates unique and unambiguous prefixed names that are intended to be interpreted as part of a Turtle document. To do this, the algorithm constructs the local part of a prefixed name by joining symbolic names in the modelling language with the ‘:’ character, and prepending the empty prefix, ‘:’. This means that we must make the requirement that the empty prefix be defined for this use. Using this algorithm, a reference for the y internal state of site site2 of agent A is derived from A(site1^∼^u^∼^v,site2^∼^x^∼^y^∼^z) as :A:site2:y. Since the empty prefix being defined to some base URI for the model file, this is a globally unique reference to that particular state of that particular site and can then be used to composed unambiguous URIs.

Although most of the entities in rule-based modelling languages possess symbolic names, rules do not. In Kappa, each rule can be preceded by free text surrounded by single quotes. To give the rule a name, we require that this free text is conformant with the local name syntax in Turtle and SPARQL (http://www.w3.org/TR/rdf-sparql-query) languages. Identifiers for subrules are created by adding their position index, based on one, to the identifier for a rule (Fig. 4B).

## 3 An annotation ontology for rule-based models

Ontologies such as GO, SBO and controlled vocabularies such as BioModels.net qualifiers have already been widely adopted for the annotation of quantitative models (Juty *et* *al.*, 2013). BioModels.net qualifiers are formed of *model* and *biology* qualifiers. The former offers terms to describe models. Examples include is to link a model to a model repository and isDescribedBy to capture information about the publication where a model has been described. The latter provides terms to map entities in a model to biological concepts. Examples include is to describe a modelling entity and hasPart to describe parent-child relationships. In addition, SBO provides a number of terms about biochemical parameters. The BioModels.net qualifiers are also ideal to annotate rule-base models, but additional qualifiers are needed to fully describe rule-based models. These are specific to the annotation of rule-based models and so we define a distinct ontology – the *Rule-Based Model Ontology* – in the namespace http://purl.org/rbm/rbmo# conventionally abbreviated as rbmo though for brevity we omit the prefix in this text if there is no risk of ambiguity. Each qualifier is constructed by combining this namespace with an annotation term. A subset of significant terms is also listed in Table 1 and the full ontology is available online at the namespace URI.

**Table 1.** Selected rbmo ontology terms for representing rule-based models

Term	Description
Kappa, BioNetGen	Model types
Agent	Type for declarations of biological entities
Site	Type for sites of Agent s
State	Type for internal states of Sites
hasSite, hasState, siteOf, stateOf	Predicates for linking Agents, Sites and States
Rule	Type for interactions between agents
hasSubrule, subruleOf	Specifies that a rule has a subrule (i.e. KaSim subrules)
Observable	Type for agent patterns counted by a simulation

The Model classes such as Kappa and BioNetGen specify the type of the model being annotated. Declarations of physical molecules, which participate in rules, are identified with the term Agent. The Agent class can represent agents and tokens in Kappa, or molecule types in BioNetGen. Site and State represent sites and states in these declarations respectively. The rules are identified using Rule. The predicates hasSite and hasState and their inverses are used to link amongst agent, site and internal state declarations. Rules can also be composed of other rules, which are linked with the parent rule using hasSubrule and its inverse.

Table 1 deals with terms related to the declaration of the basic entities from which models are constructed. The terms that begin with an uppercase letter are types (in the sense of rdf:type, and also in this instance owl:Class) for the entities in the model which the modeller could be expected to explicitly annotate. The predicates begin with a lowercase letter are used to link entities to their annotations. Table 2 has terms to facilitate representation of rules in RDF. This change of representation (materialization), from Kappa or BioNetGen to RDF is something that can easily be automated, and we have produced a tool to do this for models written in Kappa. One would not like to materialize the representation of the rules by hand as it is somewhat verbose – conciseness is a virtue of these modelling languages, not of RDF – and it is not useful for simulation since the simulation tools do not understand it. It *is*, however, useful for analysis of models since it merges the model itself with the metadata in a uniform way amenable to querying. We speculate that it may also be useful as an intermediate language for transforming between modelling languages.

**Table 2.** Selected rbmo ontology terms for representing rules in RDF

Term	Description
Pattern	Type of a pattern as it appears in a Rule or Observable
lhs, rhs	Predicates for linking a Rule to its left and right hand side Patterns
pattern	Predicate for linking an Observable to the patterns that it matches
agent	Predicate for linking a Pattern and a site within it to the corresponding Agent
status	Specifies a status of a particular Site (and State) in a Pattern
isStatusOf, internalState	Predicates for linking a status in a Pattern to corresponding Site and State declarations
isBoundBy	Specifies the bond that a Site is bound to in a particular Pattern. Bonds are identified via URIs
BoundState, UnboundState	Terms denoting that a Site in a Pattern is bound or unbound

Annotations that cannot be derived from the model and so must be supplied externally are written explicitly in RDF/Turtle using the terms from Table 1 embedded in comments using a special delimiter. The model itself is written in the standard language designed for this purpose. Additional statements can then be derived by parsing and analyzing the model using terms from Table 2 and the same naming convention from the algorithm described in Section 2.3. These statements are then merged with the externally supplied annotations to arrive at a complete and uniform representation of all the information about the model.

The rbmo ontology fills a necessary gap for describing rule-based models, but on its own it is not sufficient. Fortunately the open-ended nature of the RDF data model means that it is possible to freely incorporate terms from other ontologies and vocabularies, including application-specific ones. Two such terms are of structural importance here. The dct:isPartOf predicate from DCMI Metadata Terms is used to denote that a rule or agent declaration *is part of* a particular model (or similarly with its inverse, dct:hasPart). There is likewise a need to link internal states of sites to indicate biological meaning. The bqiol:is predicate from the *Biomodels.net Biology Qualifiers* is used for this purpose. Table 3 lists useful ontologies and vocabularies with their conventional prefixes that are used to annotate of rule-based models in this paper. This list is not exhaustive and can be freely extended.

**Table 3.** Conventional prefixes for ontologies and controlled vocabularies used in this paper to annotate models

Prefix	Description
rbmo	Rule-based modelling ontology (presented in this paper)
dct	Dublin Core Metadata Initiative Terms (http://www.dublincore.org/documents/dcmi-terms)
bqiol	BioModels.net Biology Qualifiers (Li *et al.*, 2010)
go	Gene Ontology (The Gene Ontology Consortium, 2001)
psimod	Protein Modification Ontology (Montecchi-Palazzi *et al.*, 2008)
so	Sequence Ontology (Eilbeck *et al.*, 2005)
sbo	Systems Biology Ontology (Courtot *et al.*, 2011)
chebi	Chemical Entities of Biological Interest Ontology (Degtyarenko *et al.*, 2008)
uniprot	UniProt Protein Database (Magrane and UniProt Consortium, 2011)
pr	Protein Ontology (Natale *et al.*, 2011)
ro	OBO Relation Ontology (Smith *et al.*, 2005)
owl	Web Ontology Language (http://www.w3.org/TR/owl-features)
sbol	The Synthetic Biology Open Language (Galdzicki *et al.*, 2012, 2014)
foaf	Friend of a Friend Vocabulary (http://xmlns.com/foaf/spec)
ipr	InterPro (Mulder and Apweiler, 2008)
biopax	Biological Pathway Exchange Ontology Ontology (Demir *et al.*, 2010)

## 4 Adding annotations to rule-based models

**Fig. 2.**
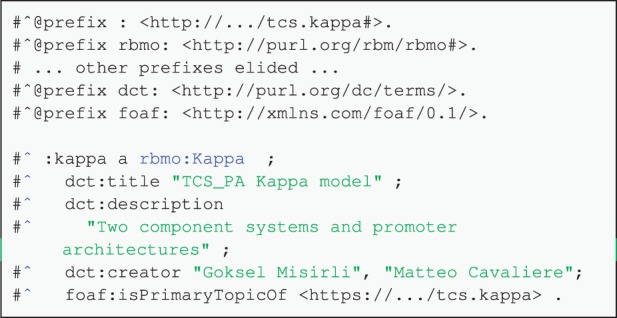
An example model annotation, with details about its name, description, creators and online repository location. All the prefix definitions required to annotate the model are also defined first, and the empty prefix is defined for the model namespace itself

Models start with a list of prefix definitions representing annotation resources providing relevant terms for the annotation of all model entities such as agents and rules. These definitions are followed by statements about the title and description of the model being annotated, using the title and description terms from *Dublin Core.* Moreover, model level annotations can be expanded to include model type, the creator, creation time, its link to an entry in a model database and so on (Fig. 2). Table 4 shows how different entities in a rule-based model can be annotated using terms from rbmo and other vocabularies.

**Table 4.** Annotating entities in rule-based models

Term	Annotation values
*Agent declarations*	
rdf:type	Agent
dct:isPartOf	Identifier for the Model
hasSite	Identifier of a Site
biopax:physicalEntity	A biopax:PhysicalEntity term, e.g. DnaRegion or SmallMolecule
bqbiol:is	A term representing an individual type of an Agent entity, e.g. a protein entry from UniProt
bqbiol:isVersionOf	A term representing the class type of an Agent entity, e.g. a SO term for a DNA-based agent
*Site declarations*	
rdf:type	Site
hasState	Identifier for an internal state
bqbiol:isVersionOf	A term representing the type of the site, e.g. A SO term for a nucleic acid-based site or an InterPro term for an amino acid-based site
*Internal state declarations*	
rdf:type	State
bqbiol:is	A term representing the state assignment, e.g. a term from the PSIMOD or the PO
*Rules*	
rdf:type	Rule
dct:isPartOf	Identifier for the Model
bqbiol:is	A term representing an individual type of a rule, e.g. a KEGG entry
bqbiol:isVersionOf	A term representing a class type of a rule, e.g. an EC number, a SO term or a GO term
subrule	Identifier for a Rule entity
lhs${}^{†}$ rhs${}^{†}$	References to the patterns forming the left and right hand side of the rule
*Observables*	
rdf:type	Observable
dct:isPartOf	Identifier for the Model
pattern${}^{†}$	References the constituent patterns
*Patterns*	
rdf:type	Pattern
ro:hasFunction	A GO term specifying a biological function
agent${}^{†}$	Reference to the corresponding Agent declaration
internalState${}^{†}$	Reference to a representation of a site’s state
isStatusOf${}^{†}$	Reference from a site’s state to the corresponding site
*Variables*	
rdf:type	sbo:SBO:0000002 (*quantitative systems description parameter*)
dct:isPartOf	Identifier for the Model
bqbiol:isVersionOf	A term representing a variable type. If exists, the term should a subterm of SBO:0000002

Terms marked with ${}^{†}$ are used for machine-generated representations of rules and patterns, and are not usually for annotating models.

**Fig. 3.**
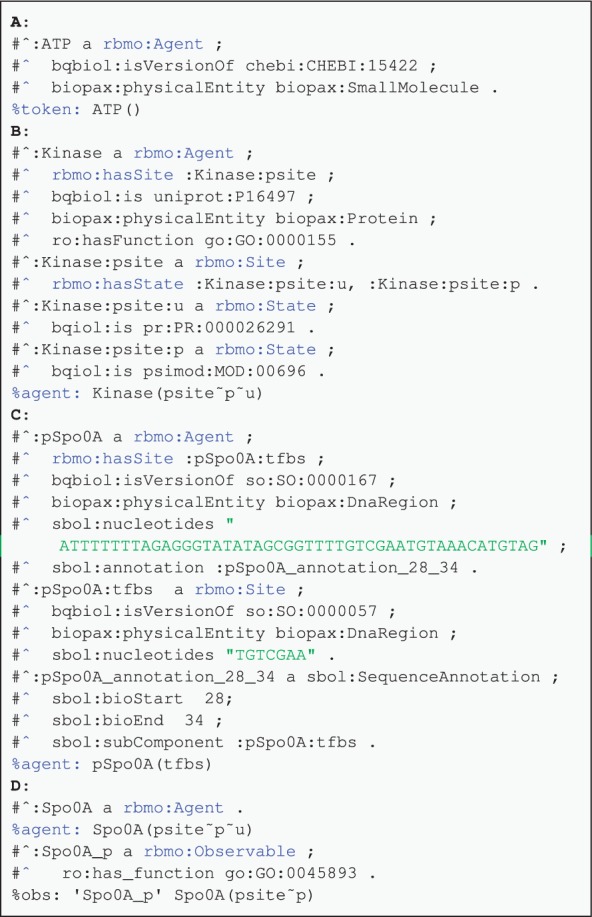
Examples of agent annotations for (**A**) an ATP token agent. (**B**) A kinase agent with phosphorylated and unphosphorylated site. (**C**) A promoter agent with a TF binding site. (**D**) An agent and an associated observable for the phosphorylated Spo0A protein, which can act as a TF

Figure 3 shows examples of Agent annotations. In Figure 3A the ATP token is annotated as a small molecule with the id of 15422 from CHEBI. Agents without sites can also be annotated similarly. In Figure 3B, the agent is specified to be a protein using the biopax:Protein value for the biopax:physicalEntity term. This protein agent is annotated as P16497 from UniProt, which is a sporulation kinase protein. It has a site with the phosphorylated and unmodified states, which are annotated with corresponding terms from the Protein Modification Ontology (Montecchi-Palazzi *et* *al.*, 2008). The ro:hasFunction term associates the agent with the GO’s histidine kinase molecular function term GO:0000155. In Figure 3C, a promoter agent with a TF binding site is represented. Both the promoter and the operator agents are of ‘DnaRegion’ type, and are identified with the SO:0000167 and SO:0000057 terms. Although the nucleotide information can be linked to existing repositories using the bqbiol:is term, for synthetic sequences agents can directly be annotated using the SBOL terms. The term sbol:nucleotides is used to store the nucleotide sequences for these agents. A parent-child relationship between the promoter and the operator agents can be represented using an sbol:SequenceAnnotation RDF resource, which allows the location of an operator subpart to be specified.

**Fig. 4.**
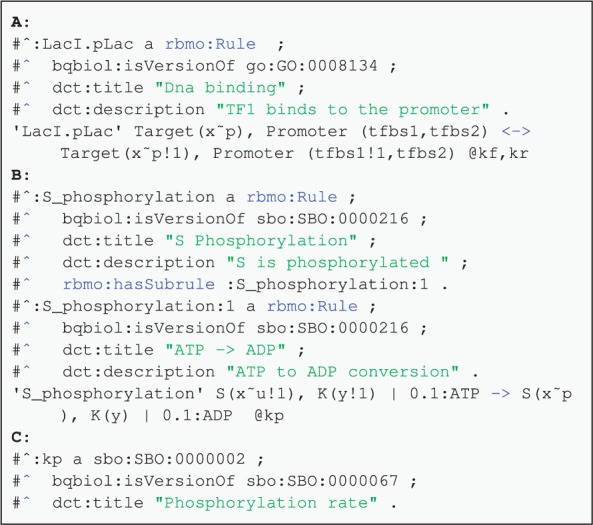
Annotating rules and variables. (**A**) TF DNA binding rule. (**B**) Phosphorylation rule with a subrule for the ATP to ADP conversion. (**C**) Annotation of a phosphorylation rate variable

Patterns can also be annotated specifically. For example, this approach could be used to annotate a pattern with a specific entry from a database. Patterns can also be explicitly stated as observables of the model. Figure 3D shows an example of such an observable. Spo0A_p represents the phosphorylated protein, which acts as a TF and is defined as an observable.

Figure 4 demonstrates annotation of rules. The first rule (Fig. 4A) describes the binding of LacI TF to a promoter. This biological activity is described using the GO:0008134 (*transcription factor binding*) term. In the second example (Fig. 4B), a phosphorylation rule is annotated. The rule contains a subrule representing ATP to ADP conversion. This subrule is linked to the parent rule with the hasSubrule qualifier. The annotation of the rate for this rule is shown in Figure 4C. Didactic fully annotated Kappa and BioNetGen models for a two-component system (TCS), controlling a simple promoter architecture are in the examples directory (files tcs.kappa and tcs.bngl in the http://purl.org/rbm/rbmo/examples directory respectively).

**Fig. 5.**
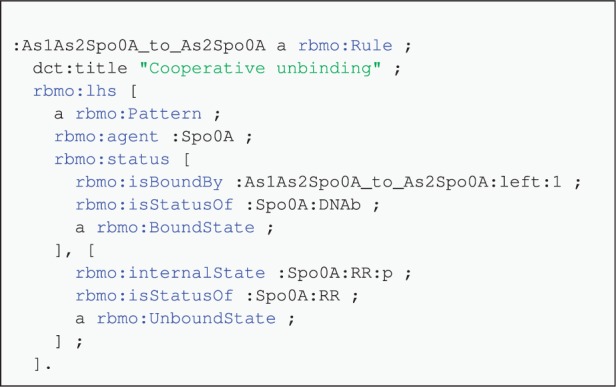
Fragment of the RDF representation of a materialized rule

Figure 5 contains a fragment of a rule materialized using our krdf tool (taken from the TCS Kappa model). The tool generates a version of the rules themselves in RDF together with the annotations. This process makes available the entire model in a uniform way that can be then used as an intermediate representation for further processing. One of the patterns involved is Sp0A(DNAb!1,RR p) which is interesting enough to illustrate the salient features. We can see that the left hand side of this rule contains a pattern involving :Spo0A and that there are two pieces of state information that are of interest. The first one refers to the :Spo0A:DNAb site, and it is bound to something (we cannot know what without the rest of the data not reproduced here). The second refers to the :Spo0A:RR site, it has a particular internal state, and it is unbound. We can also see that the rule has a title, ‘Cooperative unbinding’, which clearly could not have been derived from the rule itself. This represents a good example of merging the metadata supplied by the model author with an RDF representation of the rule.

## 5 Applications

Though development of fully functional tools is outside the scope of this paper, we demonstrate their computational feasibility and the consistency of the approach by providing some simple tools to recover and analyze the annotations embedded in a Kappa model. In particular, our proof of concept krdf tool provides enough information for simple checking of duplication of rules and inconsistencies between different parts of the model – a sort of logical type checking: these two issues are some of the basic problems encountered when composing and creating biological models (Blinov *et* *al.*, 2008; Lister *et* *al.*, 2009). Another use of this information is to draw an annotated contact map visualizing the entities involved, the interactions and the biological information stored in the annotations – this merges the classical notion of contact map used in Kappa models (Danos and Laneve, 2004; Danos *et* *al.*, 2009) with biological semantics.

The krdf tool operates on Kappa models and has several modes of operation that provide increasingly more information about the model. The first, selected with the -a option, simply extracts the modeller’s annotations and is equivalent to the unix grep command line described in the footnote on page 4. The second, selected with the -m option, *materializes* the information in the rules themselves into the RDF representation as illustrated in Figure 5. Finally the -n option *normalizes* the patterns present in the rules according to their declarations.

### 5.1 Annotated contact maps

**Fig. 6.**
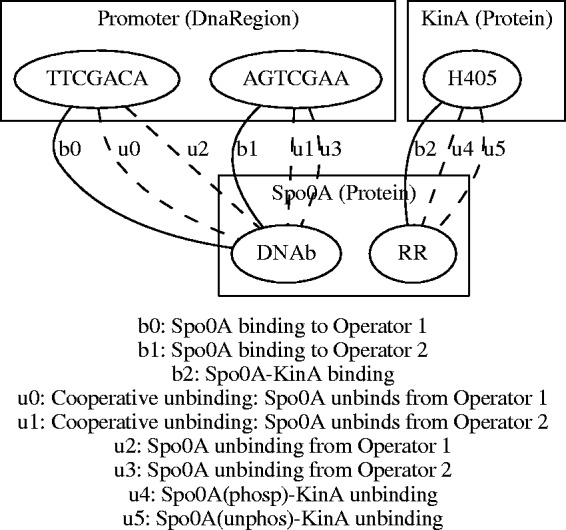
Contact map generated by a SPARQL query on the RDF materialization of the TCS example in Kappa. Biological information concerning the agents, rules and sites, types of the molecules, DNA sequences and typology of the interaction, have been extracted automatically from the model annotations

Once a complete uniform representation of the model in RDF has been generated, we can query it using SPARQL with a tool such as roqet (http://librdf.org). For example, a SPARQL query can deduce a contact map – pairings of sites on agents that undergo binding and unbinding according to the rules in the model. These pairings form a graph that can be visualized using tools such as GraphViz (Ellson *et* *al.*, 2002). Indeed with an appropriate query (See the binding.sparql file in the krdf examples directory.), roqet can directly output the result in a form that GraphViz consumes. An only slightly more sophisticated manipulation (see the contact.py script in the krdf examples directory) can extract annotations as well from the RDF representation of the TCS example model and easily create a richly annotated contact map diagram as shown in Figure 6. In this figure, biological information extracted from the annotations has been added to the agents, sites and interactions (again using GraphViz for rendering) (for simplicity, the tool assumes that only single instances of an agent are involved in a rule. However, it can be easily generalized).

### 5.2 Duplicate rule detection

**Fig. 7.**
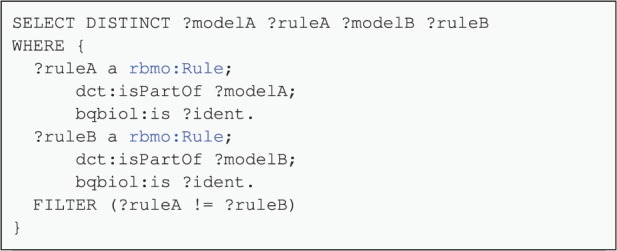
Detection of duplicate rules

One of the first tasks when combining different biological models is to detect duplicate rules. This can be done in a simple manner using the claims made about rule identity in the annotations. This approach does *not* introspect the rules to find duplicates using a sophisticated notion of equality and can be done without the need of any *α*-renaming (a renaming that would guarantee that the same symbol consistently refers to the same agent throughout the combined model). A SPARQL query such as in Figure 7 can be used on the annotations. In this case it is a join operation on the property of bqbiol:is, enforcing a stronger form of identity semantics than this predicate is usually given. The filter clause is necessary to prevent a comparison of a rule with itself. This query is a building block for model composition and illustrates the utility of annotations provided by the model author.

### 5.3 Inconsistency checking

A related query can form the basis for finding inconsistencies by using the replacement semantics of owl:sameAs. A statement of the form a owl:sameAs b means that every statement about a is also true if a is replaced by b. In particular if we have statements about the types of a and b, and these types are disjoint, the collection of statements is unsatisfiable. In other words the model has been found to be inconsistent. An OWL reasoner such as HermiT (Shearer *et* *al.*, 2008) or Pellet (Sirin *et* *al.*, 2007) will derive that a and b have type owl:Nothing in this circumstance.

To implement this work-flow we proceed as follows. First generate the fully materialized RDF version of a model using, e.g. krdf. For each use of bqbiol:is, add a new statement using owl:sameAs. Next retrieve all ontologies that are used from the web. For each external vocabulary term with bqbiol:is or bqbiol:isVersionOf retrieve a description and any ontologies that it uses recursively. Merge all of these into a single graph. This graph now contains the complete model and annotations, with entities now linked using a strong form of equality to external vocabulary terms, and we also have descriptions of the meaning of these vocabulary terms. All that remains is to ask the reasoner to derive terms that are equivalent to owl:Nothing. If there are any, an inconsistency has been identified. Furthermore using the proof generation facilities of OWL reasoners mean that given a conclusion, foo rdf:type owl:Nothing, the sequence of statements required to arrive there can be reproduced. In this way the source of the inconsistency – in the model itself, or possibly in the external resources or even the ontologies involved – can be narrowed down.

## 6 Discussion

We present an extension of rule-based models to incorporate annotations and a set of standardized terms, together with annotation guidelines, that can constitute a general proposal for annotating rule-based models. These terms can be used in a complementary manner with existing metadata resources such as MIRIAM annotations and URIs, and existing controlled vocabularies and ontologies. Such metadata is important for models that are computationally generated or served by model repositories, and opens up the possibility of using rule-based models in complex workflows. Annotations can also be used to link to human readable descriptions of models. Rules are modular and combined with annotations, can be reused in many applications.

Although, we have demonstrated the annotation of textual Kappa and BioNetGen files, our approach can be easily applied to other rule-based models. PySB (Lopez *et* *al.*, 2013) already includes a list of MIRIAM annotations at the model level, and can be extended to include the type of annotations presented here. Moreover, SBML’s multi (http://sbml.org/Documents/Specifications/SBML_Level_3/Packages/multi) package is being developed to standardize the exchange of rule-based models. The entities in this format inherit the annotation property from the standard SBML and can therefore include RDF annotations. Such SBML models can thus be imported or exported by tools such as KaSim or RuleBender in the future, avoiding the loss of any biological information. Extensions of rule-based models such as MetaKappa makes possible to define rules using abstract agents and allowing agent inheritance (Danos *et* *al.*, 2009). Modularity is especially important in synthetic biology to build complex models of intended biological systems from simple rules. The proposed schema can be easily extended in that framework.

Annotations are also useful for automated conversions between different formats. Conversion between rules and reaction networks is already an ongoing research subject (Blinov *et* *al.*, 2008), and the availability of annotations can play an important role for reliable conversion and fine-tuning of models (Harris *et al.*, 2015; Tapia and Faeder, 2013). As demonstrated above, annotations can be used to derive contact maps, which are commonly used to visualize rule-based models. Chylek and co-workers have already defined a set of glyphs to represent different nodes and edges in these graphs (Chylek *et* *al.*, 2011). This mapping is carried out by creating model guides which have contact maps enriched with information, but this process is done manually. It is straightforward to use the framework presented and automatically map agents and rules to these glyphs or to convert models into other visual formats such as SBGN or genetic circuit diagrams (Misirli *et* *al.*, 2011). Models annotated with SBOL terms can be read for subsequent analyses, for example to produce genetic circuit diagrams using standard SBOL Visual icons.

Model annotations are designed for machine readability and ideally should be produced computationally, for example by model repositories. The authors are currently developing APIs and tools to facilitate this process and in particular the access to a set of biological parts (Cooling *et* *al.*, 2010; Misirli *et* *al.*, 2014) that will incorporate rule-based descriptions and will be annotated with the proposed schema. Composing together models from these repositories requires further research, and the annotations described here can provide sufficient additional information to make the problem computationally tractable.

In general, automatic annotation of models can be challenging where the meaning of modelling entities are not known to computational tools and only the names of entities can be used to infer their semantics. This issue is an ongoing research subject and tools such as Saint (Lister *et* *al.*, 2009) and SyBIL (Blinov *et* *al.*, 2010) could be extended to automate the annotation of rule-based models. The extensive information available in biological databases and the literature can thus be integrated and made available via rule-based models, taking advantage of the syntax and the framework presented in this work.

Enriching models through computationally tractable annotations has many benefits. The computational feasibility of the proposed annotation schema has been shown with the development of a simple tool that, exporting the embedded annotations, can be used to detect duplicate rules, inconsistencies and provide contact maps annotated with biological semantics. Despite more work need to be done in this direction and challenge large biological models, these preliminary applications highlights that the proposed annotations could constitute an important step towards the automation of the model-based design and analysis of biological systems, and hence to improve the utility of rule-based models in predictive biology. In summary, the annotation framework and guidelines presented here facilitates the annotation of rule-based models, and the development of future applications for rule-based modelling.
